# High-resolution, high-throughput analysis of *Drosophila* geotactic behavior

**DOI:** 10.1242/jeb.248029

**Published:** 2025-02-20

**Authors:** Tijana Canic, Juan Lopez, Natalie Ortiz-Vega, R. Grace Zhai, Sheyum Syed

**Affiliations:** ^1^ Department of Physics, University of Miami, Coral Gables, FL 33146, USA; ^2^ Department of Molecular and Cellular Pharmacology, University of Miami Miller School of Medicine, Miami, FL 33136, USA; ^3^ Department of Neurology, University of Chicago, Chicago, IL 60637, USA

**Keywords:** Geotaxis, *Drosophila*, Multi-object tracking, Slips and falls, Kalman filter

## Abstract

*Drosophila*’s innate response to gravity, geotaxis, has been used to assess the impact of aging and disease on motor performance. Despite its rich history, fly geotaxis continues to be largely measured manually and assessed through simplistic metrics, limiting analytic insights into the behavior. Here, we have constructed a fully programmable apparatus and developed a multi-object tracking software capable of following sub-second movements of individual flies, thus allowing quantitative analysis of geotaxis. The apparatus monitors 10 fly cohorts simultaneously, with each cohort consisting of up to 7 flies. The software tracks single flies during the entire run with ∼97% accuracy, yielding detailed climbing curve, speed and movement direction with 1/30 s resolution. Our tracking permits the construction of multi-variable metrics and the detection of transitory movement phenotypes, such as slips and falls. The platform is therefore poised to advance *Drosophila* geotaxis assay into a comprehensive assessment of locomotor behavior.

## Teaser

**Summary:** When startled, fruit flies rapidly move against gravity. This natural response can be exploited to learn how a disease affects movement. An improved method is introduced to study this behavior.

## INTRODUCTION

Earth's gravity regulates the shape and size of organisms during development and continues to affect their function throughout life (Narayanan, 2023; Takahashi et al., 2021). Animals use gravity primarily for navigation and orientation, allowing them to locate food and suitable habitat. When startled, the fruit fly *Drosophila melanogaster* displays a natural tendency to move against gravity, resembling an escape response. Flies sense and orient themselves against gravity primarily through Johnston's organ in the antenna along with other sensory structures (Armstrong et al., 2006; Bender and Frye, 2009; Kamikouchi et al., 2009; Kladt and Reiser, 2023 preprint). This innate movement against gravity is called negative geotaxis and is elicited in the laboratory typically by tapping flies to the bottom of a vial and monitoring their subsequent vertical climb. The climbing assay dates back over 60 years and has been coupled with sophisticated genetic tools available in *Drosophila* to elucidate aging and general decline of motor output in disease states (Hirsch and Erlenmeyer-Kimling, 1961; Zhu et al., 2022).

Despite their widespread utility, current platforms for geotactic experiments have several limitations that can be broadly divided into those related to hardware for initiating movements and those related to software for tracking. Traditionally, the climbing behavior is visually monitored or video recorded and the number of flies reaching a certain height at the end of ∼10 s is the measure of their negative geotaxis. Though this approach provides a simple and rapid measure of geotactic response sufficient for detecting gross differences between groups, manual tapping of vials introduces inconsistencies into experiments. To reduce variability, specially designed rigs with automated and uniform force delivery have been demonstrated (Cao et al., 2017; Gargano et al., 2005; Podratz et al., 2013; Spierer et al., 2021). Data from these experiments are either scored manually or quantified using computer programs. Although more reliable than manual scoring, computer programs employed to track geotactic activity generally report population or experiment-wide average metrics such as speed and final percentage of successful climbers (Kohlhoff et al., 2011; Podratz et al., 2013; Spierer et al., 2021). While such analysis can be valuable, it overlooks individual behavioral attributes that can offer critical insights into understanding geotaxis.

Here, we present a new platform for measuring and interpreting *Drosophila* geotaxis at a single-fly, sub-second resolution, addressing most issues with currently available methods. The platform combines the strengths of a fully automated apparatus with a powerful computer vision tracking algorithm, enabling generation of data that are reproducible within the natural variations in fly behavior, while tracking provides detailed locomotor trajectories of single flies with ∼97% accuracy and a 1/30 s temporal resolution. We leverage the high-resolution tracking data to go beyond population-averaged quantities and construct multivariable analytics gleaned from individual flies and video frames to help explain geotactic behavioral differences seen in several wildtype laboratory strains.

## MATERIALS AND METHODS

### *Drosophila* husbandry

We utilized three laboratory strains in these studies: *white* (*w^1118^*), Canton-S (*CS*) and *yellow, white* (*yw*). Virgin females were separated and maintained at 25°C on standard lab food on a 12 h:12 h light:dark (LD) cycle for 5 or 6 days. The flies were then briefly anesthetized with CO_2_ and loaded into experimental vials. Experiments were conducted after flies acclimated for an hour at 25°C and 75% relative humidity.

### Hardware and video recording

We used a custom-designed acrylic vial mount that holds 10 plastic vials (Uline, Pleasant Prairie, WI, USA; model S-21972) and has an opaque plastic backing connected to a plastic rod. A metal lever, connected to a programmable stepper motor (Trinamic, model PD86-3-12978), lifts the mounted vials ∼6.5 cm by pushing up on the acrylic rod during half a rotation then lets the vials fall for the second half. A mechanical sensor (Omron Electronics Inc.-EMC Div, model SS-10GL2T) at the bottom of the lever rotation sets the start and stop positions of the motor. By default, the vials are vertically moved 4 times in 3 s, but this setting can be changed by reprogramming the motor. Each vial is normally loaded with up to 7 flies, which we found allows for high throughput as well as accurate tracking. We have tested up to 10 flies per vial with acceptable accuracy but found that in general having more than 7 flies decreases individual tracking accuracy.

White light-emitting diodes (LEDs, JOYLIT model A2835-240-60K-V24-NWP) are mounted to a metal plate 4.5 cm behind the mounted vials. Two sheets of diffuser (Rosco model R3000) are placed over the LEDs. A digital camera (ImagingSource LLC, model DMK23U445) is placed on a separate table 38 cm from the center of the opaque plastic backing. The camera is adjusted using the focus knobs until the edges of the mounted vials are at the edge of the frame. Both the camera and the motor are connected to the computer via MATLAB packages (Computer Vision, Image Acquisition, Image Processing, Instrument Control and Statistics and Machine Learning Toolboxes; third-party Instrument Control Toolbox Support Package for Keysight IO Libraries and VISA Interface).

The user initiates an experiment from the MATLAB command line by specifying the number of successive videos/trials (default, 10), duration of each video (13 s), rate of video capture (30 Hz), wait time between videos (15 s) and video names (‘exp1_’). The default values used in our experiments are included in parentheses. Once a new experiment is started, the computer engages the motor, waits 3 s for the fly vials to come to a complete stop, then begins recording the first video. Once complete, the program saves the video as an ‘.mp4’ with the appended video number, then waits the specified time to start the next trial.

### Video processing

Processing of the video recordings starts with defining the rectangular regions of interest (ROI) around the vials for each individual video. The ROI is saved in the working folder and can be utilized for recursive video processing. The saved videos are subsequently pushed through a sequence of steps that utilize the calibration data to localize, label and track each fly over time (Movie 3). Finally, the tracked fly coordinate data are used for assessing geotaxis through several analytic outputs. This pipeline of simultaneously measuring multiple groups and interpreting each group video separately offers flexibility for meta and comparative studies across trials, number of flies, treatments and genotypes.

### Calibration

The calibration step is recommended whenever a fly of significantly different size or morphology is first used, a new camera is introduced or the platform-camera distance is altered. The calibration program (Fig. S2B) presents a video frame in which boxes have been drawn around individual ‘objects’ and asks user to classify each object as ‘Is one fly’, ‘Multiple flies’ (two or more flies together) or ‘Not one fly’ (noise). The program saves two pieces of information for every classified object: pixel area and major/minor axis lengths (Fig. S2B, enlarged panel). Axis lengths are defined as the shortest and longest lines, respectively, that can be drawn from one side of an object to another. The area characterizes the size of the objects that the tracking function will look for while the axis lengths together characterize the shape of the objects. For every video recording loaded, the program automatically presents to the user every 15th frame for assessment. The calibration table helps discriminate against objects with shape or size outside the bounds of those expected for fly strains in use. Because it informs the program about the general physical characteristics of the objects of interest, calibration also adds to the versatility of our algorithm. Simply by creating a new set of calibration data, a user can examine insect strains of drastically different characteristics or under different apparatus set ups.

### Manual accuracy assessment

The tracked ROI videos and the associated coordinate matrices are used to check whether the same fly is associated with the correct track number over time. For every 15th frame, each fly is manually assessed as correct if the fly is within the appropriate bounding box. If correct, the button ‘correct’ is clicked and will add the instance to a tally. If incorrect, the button ‘incorrect’ is clicked and the program waits for the mouse click with the accurate fly location from which the Euclidean distance to the saved track is calculated and reported in a spreadsheet file.

### Calculation of metrics

#### Climbing curves

Climbing curves are constructed for a population by noting the time of the first instance each fly crosses a given height (e.g. 10 cm above vial bottom). The cumulative sum of these data generates step-like patterns with a step size of 1 when counting individuals in a single trial (e.g. Fig. 3C, left) or a step size of 1/*N*_trials_ when counting average number of individuals from *N*_trials_ (e.g. Fig. 3C, right). A fly that has crossed above a target height (maximum target=14 cm from vial bottom) is counted as having climbed to the height even if the fly displays positive geotaxis later on and goes below the target.

#### Speed and angle

We calculate the Euclidean distance moved by a fly between frames *i* and *i*+1 according to:
(1)

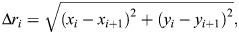
where (*x_i_*, *y_i_*) and (*x_i_*_+1_, *y_i_*_+1_) are the position coordinates in the two corresponding frames. The related speed *s_i_* is defined as Δ*r_i_*/Δ*t*, where, throughout this work Δ*t*=1/30 s. Δ*r_i_*≥4 mm are excluded, as they are considered abnormally large frame–frame locomotion. This cut-off in frame–frame displacement values prevents artifactual jumps in position, slips, falls and occasional flight within the vial from being considered in the speed–angle analysis. It is because of this constraint that our fly speed data have an upper limit of ∼100 mm s^−1^. The movement direction between frames *i* and *i*+1 is calculated according to:
(2)

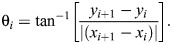
Taking the absolute value of Δ*x* makes a rightward movement indistinguishable from a leftward movement, keeping the focus on whether a movement is in the +*y* or −*y* direction. θ*_i_* is undefined if a fly is stationary between two video frames; that is, if Δ*r_i_*=0 between frames *i* and *i*+1. By counting the number of non-zero angular changes Δθ≠0, the θ*_i_* can also yield a trajectory turning rate for a cohort. For example, we find that a *w^1118^* fly makes ∼24 turns s^−1^, a *CS* fly makes ∼22 turns s^−1^ and a *yw* fly makes ∼20 turns s^−1^. It is important to note, however, that the turning rate calculated this way relates to changes in the track direction and not necessarily the heading direction. Finally, the average speed and angle are calculated from the frame–frame *s_i_* and θ*_i_*, respectively. The average metrics also readily lend themselves to other quantities as well. For instance, the average speed multiplied by the duration of video gives the distance traveled while the average angle provides a rough snapshot of a cohort's net travel direction.

#### Bootstrapping speed–angle correlations

To estimate the mean and standard error of correlations between instantaneous speed and movement angle, we followed the basic bootstrapping procedure of randomly sampling with replacement speed–angle pairs. Each sample size, *N*_sample_, was set equal to the size of the data. For instance, *N*_sample_=41,330 for *CS* flies. Speed–angle pairs within each random sample set were separated in terms of θ<0 and θ>0 and the corresponding Spearman correlation coefficients ρ_−_ and ρ_+_ and their ratio ρ_+_/ρ_−_ were calculated. This procedure was repeated 1000 times, yielding a bootstrapped probability distribution of ρ_+_/ρ_−_, as shown in Fig. 4C (right). The distribution mean and standard deviation are the ratio mean and error.

#### Extraction of analytics

Detailed guidelines on analysis of tracked videos are provided with the code library at https://github.com/sheyums/Insect-Geotaxis. Some basic familiarity with the Matlab command line is required. Briefly, the function ‘Geotaxis_analytics’ uses fly location results from tracking to calculate the metrics discussed above. The output of Geotaxis_analytics is a structure with one field – Group – itself a structure containing user-provided names and all associated data of input datasets. For example, from the analysis of four groups of data, the user can access the name and the climbing data of the 3rd group by typing ‘output.Group(3).Names’ and ‘output.Groups(3).ClimbCurves’, respectively, at the command line. Entering ‘output.Group(1).Fly_no(2).AveSpeed’ will return the average speed of the 2nd fly in the 1st group. Other results, for example the paired frame–frame speed and angular movement data of fly#19 in the 4th group, can be accessed by typing ‘output.Group(4).Fly_no(19).SpdAngle’. For additional quantification, such as determination of the number of turns made by fly#19 in group#4, the indexing feature in Matlab can again be utilized to calculate the number of non-zero angle changes between successive frames.

## RESULTS

### Mechanical design

**Fig. 1.**
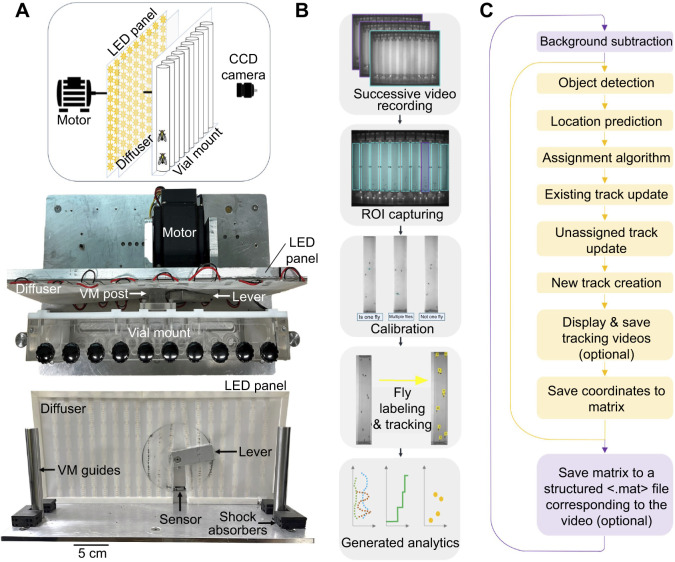
**Overview of mechanics and software for the automated system.** (A) Schematic diagram (top), overhead view (middle) and frontal view (bottom) of the mechanical device with essential components labeled on the images. VM, vial mount. (B) Flow diagram of the main steps for this assay. (C) Flow diagram detailing internal steps of the motion-based, multi-object tracking algorithm. Steps in purple are repeated for each vial and steps in yellow are repeated for each frame within a vial video.

To reduce commonly found systematic errors in manual assessment of fly geotaxis, we built an apparatus (Fig. 1A) that delivers a fixed number of taps of identical force over the same duration across experiments. The core of the apparatus consists of a custom-designed acrylic vial mount (VM) that holds up to 10 plastic vials with slots on the side that allow it to slide vertically along metal guides (VM guides). When the motor is activated, a metal lever connected to it contacts the VM post, lifting the VM for the first half of the lever's rotation and allowing the VM to fall during the second half. A mechanical sensor (Fig. 1A, bottom panel) detects lever motion and provides an appropriate signal to initiate or terminate motor control. Normally, the VM is vertically moved 4 times in 3 s, ensuring all flies are at the bottom of their respective vials before video recording starts. A panel of LEDs provides diffuse illumination from behind the VM.

### Video recording and image processing

A CCD camera acquires grayscale images at a rate of 30 Hz once the vial mount comes to rest. A 10 s recording (Movie 1) constitutes one trial. The process of mechanical fly perturbation and subsequent movement recording is usually repeated 10 times per experiment (Fig. 1B). Each video is cropped by ROI and saved as separate videos (Fig. 1B; Movie 2). A subset of ROI videos can be used for calibration, where the user manually classifies detections into categories of single and multiple flies in different orientations and distances from the camera. The calibration step is critical as it yields shape and size distributions of flies as imaged by the CCD and helps the tracking algorithm (see Materials and Methods) reject any non-fly objects captured.

### Tracking summary

Tracking individual flies (Movie 3) over time is the most challenging task for our method and we delve into its details over the next three figures. In brief, the first step in our tracking is the creation of a background frame (Fig. 1C). Objects are found by thresholding background-subtracted individual frames. Among detected objects, only those falling within shape and size distributions from calibration data are accepted as flies. A series of specialized algorithms next predicts the flies' locations and assigns each a track. These steps of prediction and updated assignment are repeated for each CCD frame and the position information is saved in a matrix.

### Tracking errors

The relatively unconstrained movement of flies produces four major types of tracking errors (Fig. S1) which the algorithm must rectify to achieve high accuracy. (1) Occlusion by another fly or temporary immobility can cause a fly to become invisible to tracking only to reappear frames later (Fig. S1A). This ‘lost and found error’ is addressed by updating the unassigned track with the last known coordinates and utilizing the assignment algorithm to reconnect the correct fly with its existing track once it reappears. (2) Two or more flies can appear as a single detected object by virtue of proximity. In this type of ‘grouping error’ (Fig. S1B), the enlarged size of the detected object combined with the multiple fly characteristics from calibration would be used to create overlapping tracks from a single detection. (3) Overlap of multiple flies can also lead to switching of individual assignments (Movie 3). To estimate the frequency of this error, we analyzed 79,800 video frames each with 7 flies and detected 2762 instances of fly collisions (3.5% or ∼7 overlaps per 200 frames). But as swapping of labels in a collision is a random event, we assume about half of collisions lead to identity switching. In other words, we incur an identity switch in <2% of video frames (<6 switches per 300 frame video). Importantly, identity switch does not cause loss of single-fly resolution. Because our analyses rely on anonymized single-fly data, the less than 0.6% error (<12 incorrect values within a 7×300 location matrix) contributed by these events is negligible in the inherently heterogeneous behavioral metrics we compute. (4) Another source of assignment-related error is aberrant movement – stationarity and unexpected movement direction. For instance, two flies that are separate from each other can erroneously exchange their assignments (Fig. S1C), or preference is incorrectly given to an aberration in illumination instead of a fly, if the aberrant object is more mobile than the fly (Fig. S1D). However, the large unphysical jumps in positions can be used to correct both types of error.

### Background subtraction and detection calibration

**Fig. 2.**
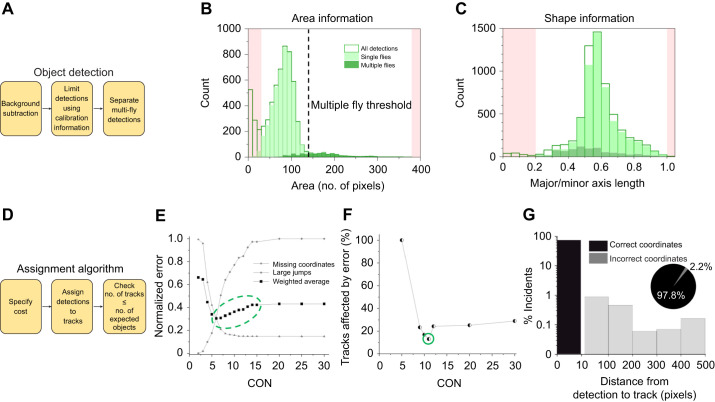
**Using calibration information and cost calculation for optimizing assignment.** (A) Flowchart of the main detection steps. (B,C) Histograms of pixel areas (B) and the ratio of minor to major axis lengths (C) of all detected objects. Shaded regions at histogram extrema correspond to noise detection. The legend in B applies to B and C. (D) The main steps in detection-to-track assignment. (E) Computational assessment over 17 cost of non-assignment (CON) values. The average number of untracked frames is normalized to the maximum average found (triangles). The top 1% of the largest frame-to-frame displacements of each track are averaged and normalized to vial length (diamonds). The weighted average (squares) uses coefficients of 1 and 0.1, respectively. The green oval highlights a CON range yielding greater accuracy. (F) Manual assessment of tracks affected by one or more errors over a narrower CON range identifies CON=11 as the most optimal (green circle). (G) Histogram of fly location error (in pixels). Inset: percentage of flies localized to <10 pixels. The same 50 videos (256 flies) were used in B,C and F,G.

Detection of flies in each image frame can be broadly divided into three steps (Fig. 2A): (a) background subtraction, (b) applying size and shape discrimination to identify true flies among detected objects, and (c) determining whether one or more flies comprise each detection. A background frame is generated for each vial by calculating the median pixel value from all frames. Each frame is subtracted from the background and thresholded by setting pixel values ≤0.06 to 0 and the rest to 1 (Fig. S2A). Because several flies are moving in three dimensions through an imperfectly illuminated space, the detections after background subtraction can include single and multiple flies and noise objects such as shadows and streaks. To correctly account for all flies, the user can opt to go through calibration (see Materials and Methods).

Fig. 2B,C shows calibration data for a set of several commonly used wildtype strains (*w^1118^*, *CS*, *yw*) as examples. Objects with areas larger than the maximum of the multi-fly areas and smaller than the lowest 16% of single-fly areas are classified as noise in our algorithm (Fig. 2B, shaded regions). There is some overlap between the single- and multi-fly area distributions and to remove ambiguity we define the maximum single-fly area to be the threshold for multiple flies (Fig. 2B, dashed vertical line). Therefore, objects are accepted as multiple fly detections if their areas are larger than this threshold and smaller than the maximum multi-fly area. We arrived at these cut-offs through trial and error aimed at maximizing fly identification accuracy. However, the extreme cut-offs alone do not eliminate all false positives as the left tail end of the single-fly distribution can still have some overlap with noise. Shape information, specifically the ratio of minimum and maximum axis lengths, is used to eliminate remaining incorrect detections (Fig. 2C). Objects with axis-length ratios not corresponding to single or multi-fly objects (Fig. 2C, shaded regions) are marked as noise detections and removed from further analysis.

The detections that remain after size- and shape-based discrimination are highly likely to be only flies. To account for the total number of flies, we determine the number of individual flies in the multi-fly classifications by dividing each multi-fly area by the maximum single-fly area found in calibration then rounding to the bigger whole number. Individuals found in a multi-fly object are given a common set of coordinates and appended to the list of detections. Though these steps do not guarantee every fly in the frame is detected, they do ensure that every detection is an individual fly.

As our goal is not limited to detecting flies but also involves following them over time, the algorithm next associates the detected fly locations in one frame with fly locations from the previous frame. The association is done by assigning each detection to the most likely track, through a scheme called the Hungarian matching algorithm (Munkres, 1957). In our method, we complete the detection-to-track matching in three steps (Fig. 2D). The first task is constructing the cost matrix (Fig. S3) whose columns represent locations in the current frame (Detections) and whose rows represent locations from the previous frame (Tracks). For each element of the cost matrix, the Euclidean distance between a current detection and its predicted location is calculated, *d*_pred_. For predictions, we use the constant velocity Kalman filter. The filter first makes an initial guess about the future location of a fly using default inputs and updates the guess after comparing it with the final assigned location. However, we found that a cost matrix relying only on Kalman filter predictions is unreliable for our experiments because of idiosyncrasies in fly movement. We therefore define matrix elements as:

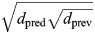
where *d*_prev_ is the Euclidean distance between current and previous detection coordinates. As detection relies on motion, stationary individuals could be lost, or it may take several frames to recognize a fly. The solution for either is the creation of ‘phantom’ tracks and detections within the cost matrix to allow for lost or new flies (Fig. S3, tracks 1 and 3).

In creating phantom tracks, the cost matrix is padded with an experimentally determined value called the cost of non-assignment (CON). The CON is a threshold value that determines whether assigning a detection to a track is too costly. If the CON is too low, it will not assign detections to tracks, resulting in missing coordinates or ‘lost and found errors’ and if the CON is too high, it will assign detections to the wrong tracks, resulting in swap and jump errors. To visualize the effect of the CON, we recorded the number of missing coordinates and the number of abnormally large jumps in coordinates recorded, as we ran the tracking algorithm on a set of 50 videos at 17 different CON settings (Fig. 2E). As expected, increasing CON minimizes the number of missing coordinates but increases bias towards detection of large location jumps. The optimal CON produces a minimum in their average. As large jumps included in this estimation do not distinguish between actual falls and erroneous jumps in location, we calculated a weighted average by assigning the missing coordinates error twice the weight of the large jumps error. The weighted error gave us a narrow range of ∼6–13 to optimize the CON (Fig. 2E). Using a set of 50 videos tracked at 7 different CON, we manually assessed errors within each individual track (Fig. 2F). An entire track was considered incorrect if any significant error was found, regardless of how accurate the rest of the track was. For ease of comparison across groups, we normalized the error to the number of total tracks assessed. These studies found a clear minimum for CON=11, which we used as the default in our tracking algorithm.

If the number of tracks in a frame is found to exceed the number of flies after completion of the detection-track assignments, we recheck the distance between the detected coordinates and the previous coordinates of the unassigned tracks and remove the detection with the maximum distance. Though this accounting method is somewhat arbitrary, extra tracks are rare and do not lead to major information loss. If the number of tracks is less than the known number of flies, nothing is done as some flies remain stationary. At the end of the tracking, a random coordinate at the bottom of the vial is selected and appended to the final coordinate matrix to account for the missing fly.

To gauge the overall accuracy of our algorithm, two individuals separately evaluated tracking results of each fly in a set of 50 videos. If a tracking result was deemed inaccurate, the individual clicked on the actual fly coordinate and the distance between the actual location and the tracked location was recorded. These assessments showed that our algorithm correctly tracks flies ∼97% of the time (Fig. 2G, inset) with a positional error of <9.7 pixels≈2 mm (Fig. 2G).

### Introduction of new metrics

**Fig. 3.**
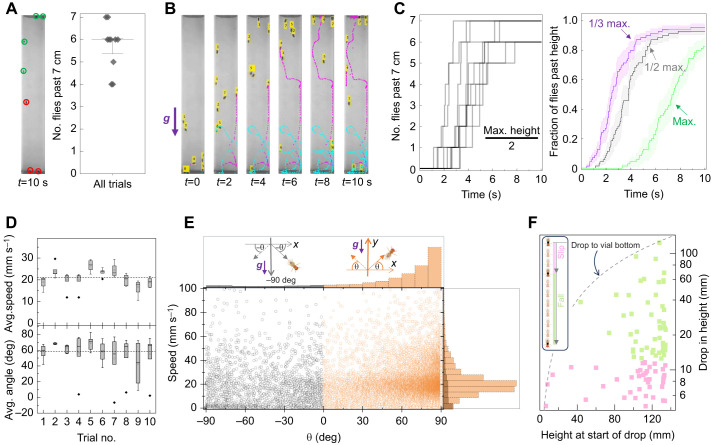
**High-resolution quantification of *w^1118^* geotactic behavior.** (A) Left: vial image showing fly (*N*_flies_=7) locations at *t*=10 s. Flies past the 7 cm height mark are circled in green; remaining flies are circled in red. Right: final fly count (median±s.e.) above 7 cm from each trial (*N*_trials_=10). (B) Time-lapse images showing tracked flies every 2 s in one trial. Yellow flags represent numbers assigned to each fly at that instant by the tracking program. Example tracks 4 and 6 over time are shown in magenta and cyan, respectively. (C) Left: individual *w^1118^* climbing past half the vial height. Each gray staircase represents a single trial. Right: average climbing curves for 1/3, 1/2 and maximum height. Mean (solid line) and s.e.m. (shaded band) for each height are from 10 trials. (D) Per-trial average speed (top) and average angle of movement (bottom). Box plots show mean (open squares), median, upper and lower quartiles, 1.5× interquartile range and outliers (filled diamonds). Dashed horizontal line denotes average across trials. (E) Scatter plot of speed and angle of movement (θ) between consecutive video frames, from all flies and trials (open circles, *N*_total_=15,422), and relative frequency histograms of speed (right) and angle (top). Data for θ>0 are in orange and those for θ<0 are in gray. Upper quadrant angles are defined as θ>0 and lower quadrant angles are θ<0 (top, cartoons). (F) Height of a fly just prior to a drop versus the size of the vertical drop (squares). Dashed line: maximum possible drop from a given height. Note: the *y*-axis is on a log scale. Inset: a ‘slip’ or ‘fall’ in terms of body length. In B,E, ‘***g***↓’ indicates the direction of gravitational force.

Traditionally, geotaxis experiments report the percentage of flies that successfully pass a threshold height after a specified time. In an experiment with *w^1118^* flies, simple counting at *t=*10 s shows that in 70% of trials, six or more flies are past the 7 cm mark, half of the full vial height (Fig. 3A). Our detailed tracking provides a more comprehensive picture of the events that led up to the end of one trial (Fig. 3B–F). Select video frames from one trial shows fly #1 (purple trajectory) reaching the top by *t*=8 s but falling to ∼2/3 of the vial height thereafter, while fly #6 (cyan trajectory) reaches <1/3 of the vial height by the end (Fig. 3B). Manual analysis would incorrectly exclude fly #1 from having displayed robust negative geotaxis before falling. Our method avoids such errors by monitoring fly positions throughout the experiment. We can construct a climbing curve for each trial with discrete steps representing the passage of single flies for a height of 7 cm (Fig. 3C, left). These data show that 100% of trials ended with 6 or 7 flies successfully passing the 7 cm height threshold, in notable contrast to traditional assessment (Fig. 3A). This discrepancy underscores a critical shortcoming of assessing climbing from the final video frame. Without knowledge of entire trajectories, recorded behavior is susceptible to brief events such as falls that can introduce significant errors. Our results show that detailed automatic tracking not only is convenient but also leads to more accurate assessment of climbing behavior.

The single-fly resolution of the staircase data also highlights individual variability generally disregarded in existing analysis methods. Although all trials ended with similar fly numbers (6 or 7) above threshold, each took a distinct path largely owing to behavioral variability in individuals. To assess the degree of variability, we averaged staircase data for three different heights across 10 trials (Fig. 3C, right). We found that the mean variation is 13%, 18% and 28% for climbing to 1/3, 2/3 and the full 14 cm height, respectively. The between-trial variability increases with height because fewer flies climb to greater heights. To assess whether there are any general trends in performance, such as degradation in climbing ability due to fatigue (Bazzell et al., 2013; Damschroder et al., 2018), we next examined the average speed and angular direction of the group in each trial (Fig. 3D). These data, which include movements in all directions, show no particular trend from the first to the last trial, indicating that fly movement kinetics do not change dramatically over the course of an experiment. We measured the angle of movement θ relative to the horizontal direction (±*x*) where θ is considered positive if it is in the upper quadrants and negative if it is in the lower quadrants (Fig. 3E, top).

While useful for learning about overall trends in geotactic kinetics, population averages can mask short time-scale dynamics and the relationship between speed and angle between consecutive frames (Fig. 3E). For the experiments on *w^1118^* flies, we found ∼89% of events have θ>0 (Fig. 3E, top histogram, orange) with an associated speed of 21.9±12.7 mm s^−1^ (mean±s.d.). For the remaining ∼11% of movements with θ<0, the associated speed distribution has a smaller average and a larger variance, 19.2±17.8 mm s^−1^ (Fig. 3E, right histogram). Though they yield similar mean values, speed histograms in the two directions assume different shapes – a peaked distribution for θ>0 and a relatively even distribution for θ<0. Differences in distribution shapes suggest the possibility of differences in how movement speed and angle are correlated, with the narrower distribution in the +*y* direction implying a bias towards negative geotaxis, consistent with the strong climbing behavior displayed by these flies (Fig. 3C). Calculation of the Spearman correlation further supports the idea of a directional bias, with a correlation coefficient of ρ_+_=0.145 (*P*<10^−60^) in the +*y* direction and a correlation coefficient of ρ_−_=0.039 (*P*=0.09) in the −*y* direction. Robustness of negative geotaxis can depend on such directional bias in movement, and these analyses indicate we can use the ratio ρ_+_/ρ_−_ as a new metric to assess the behavior.

Close examination of videos revealed that flies can occasionally lose traction and consequently slip or fall (‘drop’) during climbing. How often and how far they drop can impact their geotactic performance. To better characterize these disruptions in climbing, we defined a vertical drop −Δ*y* between two consecutive frames of more than a body length and up to three body lengths (4.5 mm≤−Δ*y*≤12 mm) as a slip. A larger vertical drop, −Δ*y*>12 mm, is defined as a fall. In the *w^1118^* cohort, we detected a total of 41 slips and 52 falls in 10 trials, indicating that <0.5% of movements involved these large drops in height. Both slips and falls occurred at various heights, with a higher incidence rate above a height of ∼8 cm (Fig. 3F). The increase in the incidence of slips and falls with height is consistent with the idea that the longer flies are engaged in climbing, the greater their chances of losing traction. Importantly, these data suggest that similar to the ratio ρ_+_/ρ_−_, slip and fall characterization can be used as an independent measure of locomotor coordination in geotaxis.

### Comparison of genotypes

**Fig. 4.**
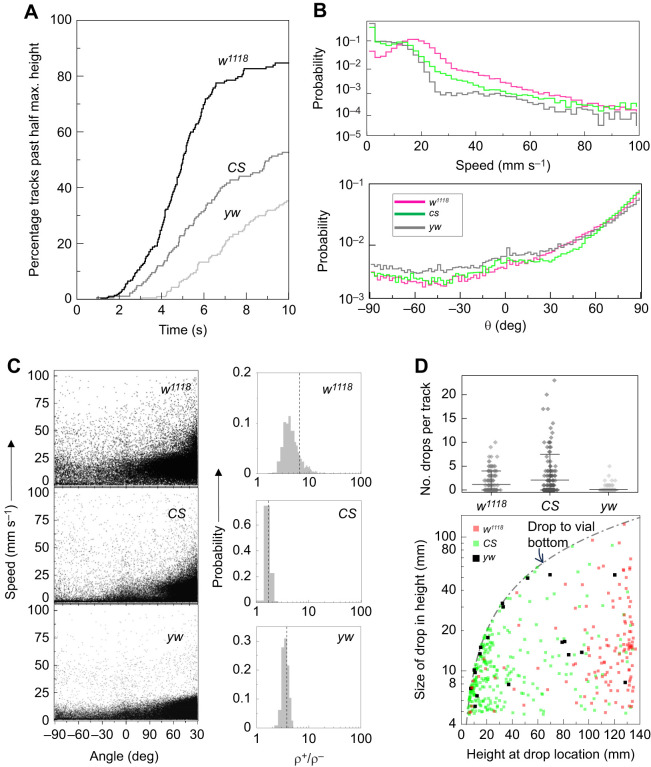
**New method identifies locomotor features underlying geotactic differences among *w^1118^*, *CS* and *yw* strains.** (A) Average climbing curves of 10 trials for *w^1118^* (*N*_flies_=18), *CS* (*N*_flies_=17) and *yw* (*N*_flies_=21) female flies. (B) Probability distributions of speed (top) and movement angle (bottom). Mean speed is 17.5 mm s^−1^ (from *n*=48,186 values), 9.5 mm s^−1^ (from *n*=48,182 values) and 6.9 mm s^−1^ (from *n*=59,845 values) for *w^1118^*, *CS* and *yw* flies, respectively; *P*<10^−200^ in all three pair-wise comparisons. Mean angle for *w^1118^* and *CS* flies is 53.6 deg while that of *yw* is 43.6 deg. The *yw* distribution differs significantly from that of the other two groups (*P*=2×10^−10^). (C) Speed versus angle scatter plot (left) and ratio ρ_+_/ρ_−_ of Spearman correlations (right) for each genotype. Bootstrapped histograms yield mean values (dashed vertical lines) of 6.6±21.1, 1.7±0.17 and 3.7±0.55 for *w^1118^*, *CS* and *yw* flies, respectively; *P*<10^−200^ in all three pair-wise comparisons. (D) Top: number of slips or falls detected per track (circles, from all trials). *yw* versus *w^1118^*, *P*=6×10^−10^; *yw* versus *CS*, *P*=1.2×10^−14^; *w^1118^* versus *CS*, *P*=0.29. Mean, 10% and 90% of data are indicated. Bottom: height of fly versus drop size. *P*-values in B–D are from Kruskal–Wallis null hypothesis test followed by a *post hoc* test.

To further exploit the analytic capabilities of our platform, we next compare geotaxis of three widely used laboratory strains *w^1118^*, *CS* and *yw*. Each genotype was measured in three independent experiments (Fig. S4). Our experiments showed that the strains differ in their geotactic performance, with *w^1118^* on average showing the best and *yw* the worst climbing ability (Fig. 4A). We wondered whether our analyses could reveal kinetic details behind these differences. Speed distributions (Fig. 4B, top) show that *w^1118^* flies have a significantly higher probability (*P*=0.31) of moving at speeds >20 mm s^−1^ compared with *CS* (*P*=0.09) and *yw* (*P*=0.03) flies. While *CS* and *yw* distributions roughly overlap for speeds <20 mm s^−1^, *CS* flies are more likely than *yw* flies to move at higher speeds. These speed data yield median values of 16.3, 7.8 and 5.2 mm s^−1^ for *w^1118^*, *CS* and *yw*, respectively, in accordance with their relative ranking in climbing rates. Movement angle distributions (Fig. 4B, bottom) showed that all three genotypes generally move in the +*y* direction (*P*≥0.81). However, *yw* flies have a slightly higher tendency to display positive geotaxis, as their probability for all movement angles θ≤0 is consistently higher than that of the other two genotypes. In addition to speed, the number of turns during walking can serve as an alternative window into locomotor activity (see Materials and Methods).

Data on speed–angle correlations indicate that *w^1118^* flies have the largest bias in the +*y* direction with a mean ρ_+_/ρ_−_ of 6.6, followed by *yw* flies with a ρ_+_/ρ_−_ of 3.7 and, lastly, *CS* with a ρ_+_/ρ_−_ of 1.7 (Fig. 4C). The large correlation bias for *w^1118^* flies is consistent with their superior climbing rate. But a larger *yw* bias compared with *CS* flies is surprising, as *yw* flies perform poorly in overall climbing. The *CS*–*yw* difference suggests that a poor ρ_+_/ρ_−_ ratio is not a major impediment to climbing ability in *CS* relative to *yw*. We also compared the incidence rate of slips and falls in the three groups. The slips and falls data show that *yw* flies have the lowest rate, ∼11 per 100 tracks on average, compared with ∼117 per 100 tracks in *w^1118^* flies and ∼200 per 100 tracks in *CS* flies (Fig. 4D, top). Thus, based solely on the drop rates, *yw* flies would be expected to be the best climbers and *CS* or *w^1118^* flies the worst. Examination of the drop locations and magnitudes provides additional insights (Fig. 4D, bottom). These data show that the majority of *w^1118^* drops occur >110 mm above the bottom, while the majority of *CS* and *yw* drops occur below ∼50 mm from the bottom. As *w^1118^* flies drop from greater heights, it is reasonable to expect the size of their drops to be larger. Yet, we find that *w^1118^* and *yw* flies have on average the same drop size of ∼20 mm while those of *CS* flies are statistically smaller, ∼12.6 mm (Fig. S5A). When the size of a drop is compared with the maximum possible size of the drop (this ratio is 1 along the curved dashed line in Fig. 4D), we find that it is 0.22, 0.53 and 0.62 for *w^1118^*, *CS* and *yw* flies, respectively; that is, a larger fraction of *yw* drops bring the flies to the bottom of their vial (Fig. S5B).

In short, we find that the two best predictors of *w^1118^*, *CS* and *yw* relative climbing performance are speed and the size of drop compared with its maximum possible size. The other metrics we introduced – such as movement angle, ρ_+_/ρ_−_ ratio and the frequency of drops – only partially capture relative geotaxis ability of the three strains. For instance, ρ_+_/ρ_−_ results correctly predict that *w^1118^* flies are the best climbers but incorrectly predict that *yw* flies are superior to *CS* flies. The incongruent conclusions drawn from the different metrics indicate that they capture different features of locomotion and are, at least partly, independent of each other. Having several independent metrics is a powerful analytic resource whose potential for uncovering geotactic aberrations will become clearer through future quantitative studies (Lobato et al., 2024).

## DISCUSSION

The custom tracking and analyses programs and detailed instructions on how to use them are freely available from GitHub (https://github.com/sheyums/Insect-Geotaxis)*.* This work joins several recent efforts to develop platforms for robust examination of fruit fly geotactic behavior. Our new apparatus builds upon prior experimental procedures by incorporating mostly off-the-shelf components in an intuitive design that allows stable, high-throughput and reproducible measurements. Computer control of the apparatus permits changes in experimental parameters. We have shown that single-fly trajectory reconstructions yield both accurate climbing curves and a wealth of kinetic metrics, enabling a deeper understanding of geotactic behavior than what has been possible thus far.

Tracking multiple freely moving flies over time is the most challenging part of single-fly trajectory reconstructions. We addressed the challenge with the application of two noteworthy computer vision tools. One is a predictive algorithm, called the Kalman filter, which provides an estimate of the most likely match between a new detection with existing tracks. We use the Kalman estimate together with a separate estimate based on previous detection coordinates. The joint estimate provides a key input for the second important computer vision algorithm, called the Hungarian method, that optimizes detection-to-track association.

The number of flies that climb to a given height within an allotted time is the most used measure of geotaxis. Current platforms with limited tracking ability report final fly count, low time resolution intermediate counts or climbing curves for only single flies per vial (Gargano et al., 2005; Podratz et al., 2013; Willenbrink et al., 2016). Other methods with ostensibly better tracking still do not present climbing curves but instead rely on other population-wide metrics (Cao et al., 2017; Damschroder et al., 2018; Kohlhoff et al., 2011; Spierer et al., 2021). Our algorithm can track multiple flies simultaneously ∼97% of the time with millimeter accuracy. The climbing curves in our studies provide a dynamic picture, revealing single-fly movements with a 1/30 s temporal resolution. The high-resolution tracking allows us to dissect geotactic behavior at the level of single video frames, demonstrated by quantifying instantaneous movement angle and speed and by conducting meta-analysis on their correlations across three different laboratory strains. Flies are known to lose traction occasionally during climbing, but prior *Drosophila* studies overlooked these events. Quantification of such falls is critical, especially in fly disease models (Chaudhuri et al., 2007; Zhu et al., 2019), as several human neurological disorders associated with loss of balance show an increased frequency of falls (Bloem et al., 2001; Stolze et al., 2004). We demonstrate that accounting for these rare events can uncover the nature of movement deficiencies underlying defective geotaxis.

## Supplementary Material

10.1242/jexbio.248029_sup1 Supplementary information
