# Supplementary material for: High-resolution, high-throughput analysis of Drosophila geotactic behavior
Source: J Exp Biol. 2025 Feb 20;228(4):JEB248029. doi: 10.1242/jeb.248029 (PMC11883285; doi:10.1242/jeb.248029)
Supplement: Supplementary information [file jexbio-228-248029-s1.pdf]

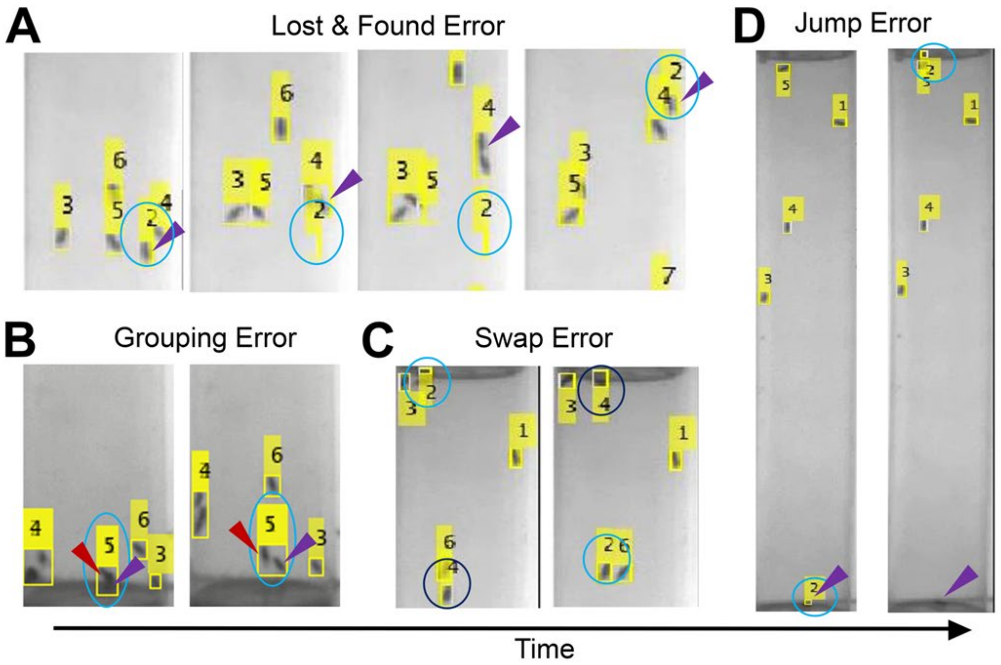

Fig. S1. Frequently occurring tracking error examples. (A) Four frames of the same video captured within 0.5 second period show how a fly disappears owing to proximity with another and reappearing once they separated. The cyan circle indicates where the track is located, while the purple arrowhead indicates the true location of the fly at each timepoint. (B) Two frames of the same video captured within 0.1 second show how two flies can be mistaken as one detection if they are too close in proximity. The cyan oval indicates where the singular track is located, the red arrow points to fly A, the purple arrow points to fly B at each timepoint. (C) Two frames of the same video captured within 0.3 second show how two tracks can switch between two flies even if they are not proximal. The cyan circle indicates track 2, the navy circle indicates track 4 at each timepoint. Note, the flies are not moving very much between these two frames. (D) Two frames of the same video captured within 0.1 seconds show how noise can create a false detection and force the track to jump from a stationary fly at the bottom to a moving shadow at the top. The cyan circle indicates the track while the purple arrow indicates the fly associated with that track at each timepoint.

Journal of Experimental Biology • Supplementary information

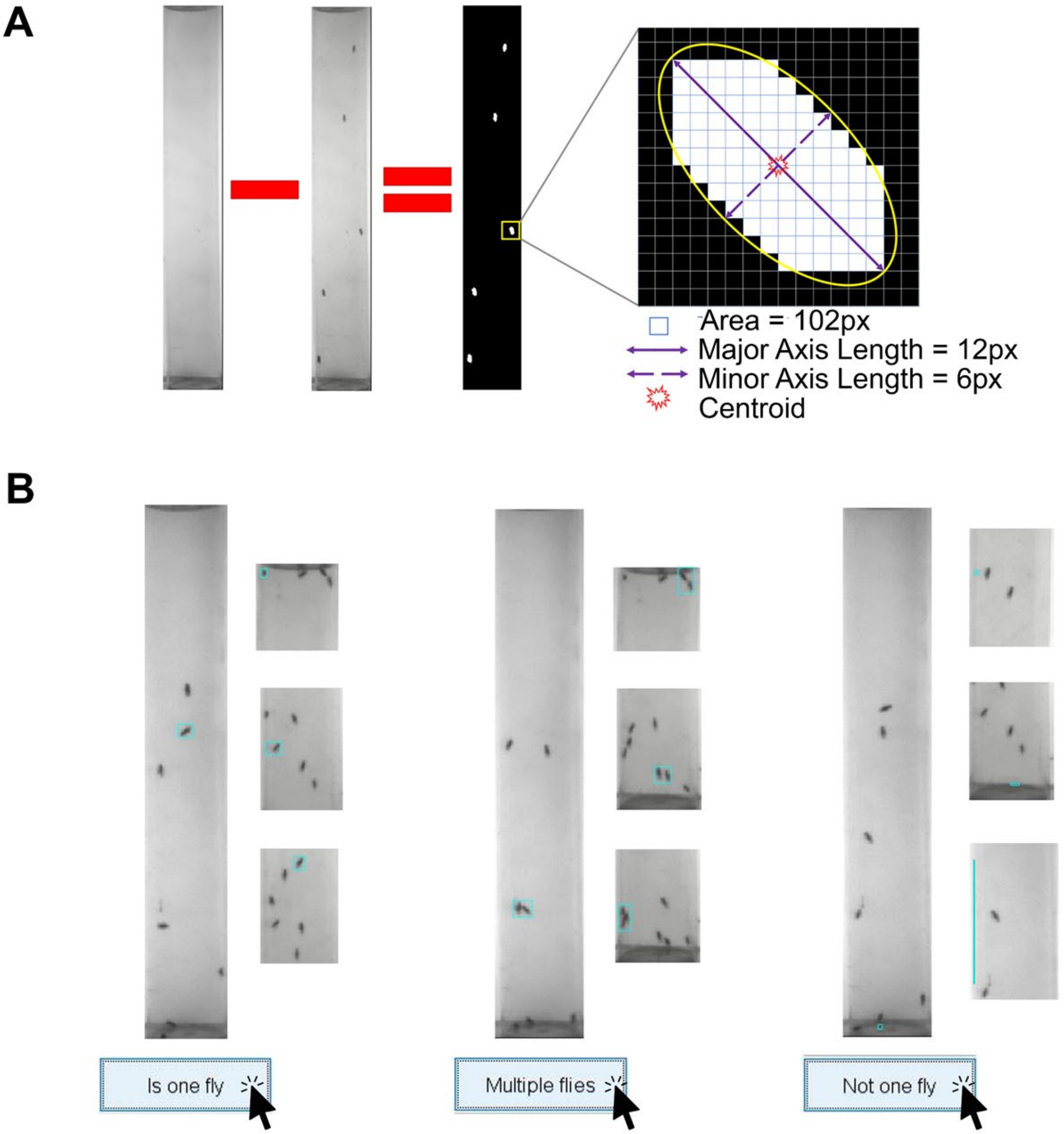

Fig. S2. An elaboration of calibration classifications for more precise object detection. (A) Visual representation of background subtraction with a zoomed in depiction of important information regarding each detection. Typical properties of a detected fly are noted below zoom. (B) Real images showcasing possible detections sorted by manual calibration. From left to right shows two columns each of singular flies per detection, multiple flies per detection, and incorrect detections (noise). Detected objects are inside cyan rectangles.

Journal of Experimental Biology • Supplementary information

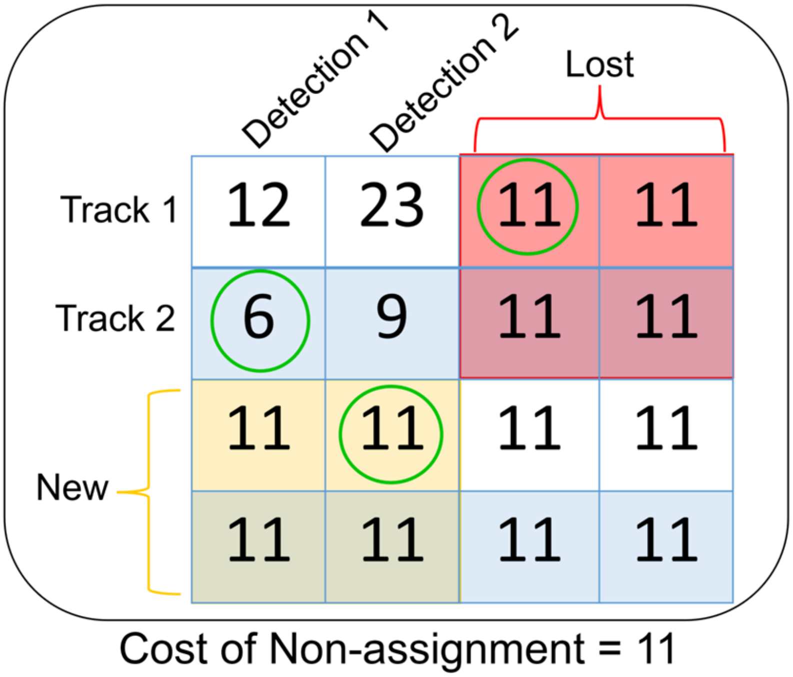

Fig. S3. An example of a padded cost matrix. Rows are tracks that existed in the previous frame of the video. Columns are detections found in the current frame of the same video. The cost matrix is padded by the cost of non-assignment to allow for new tracks to be created or lost, indicated by the yellow and red regions, respectively. Circled elements indicate the lowest total cost, thus the final assignment of the detection to track.

Journal of Experimental Biology • Supplementary information

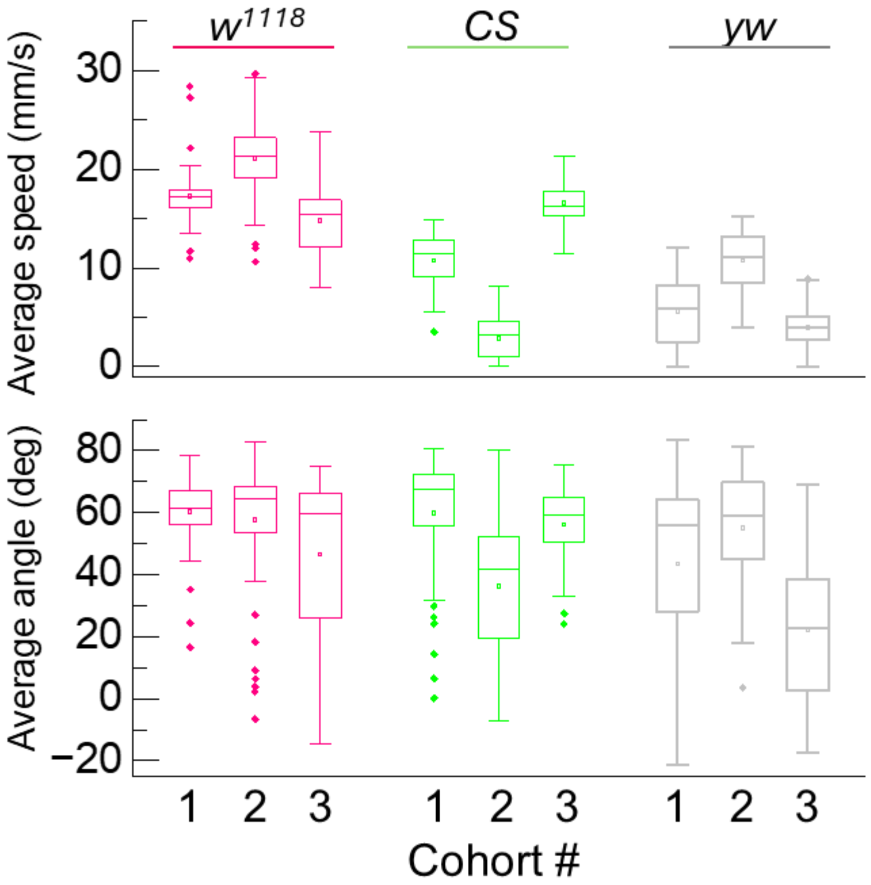

Fig. S4. Speed and angular movement data from Figure 4 averaged to illustrate experimental variability between cohorts of the same genotype. Each box plot was constructed with average speed (upper) and average angle (lower) of individual flies of a cohort. Individual averages were calculated from each trial and collated from 10 trials per cohort. Box-and-whisker plots have different representations in the literature. Here: top and bottom of box indicate 1st and 3rd quartiles, horizontal line within box indicates the median, square represents the mean, whiskers include 1.5 times the inter-quartile range, and symbols (when present) indicate outliers.

Journal of Experimental Biology • Supplementary information

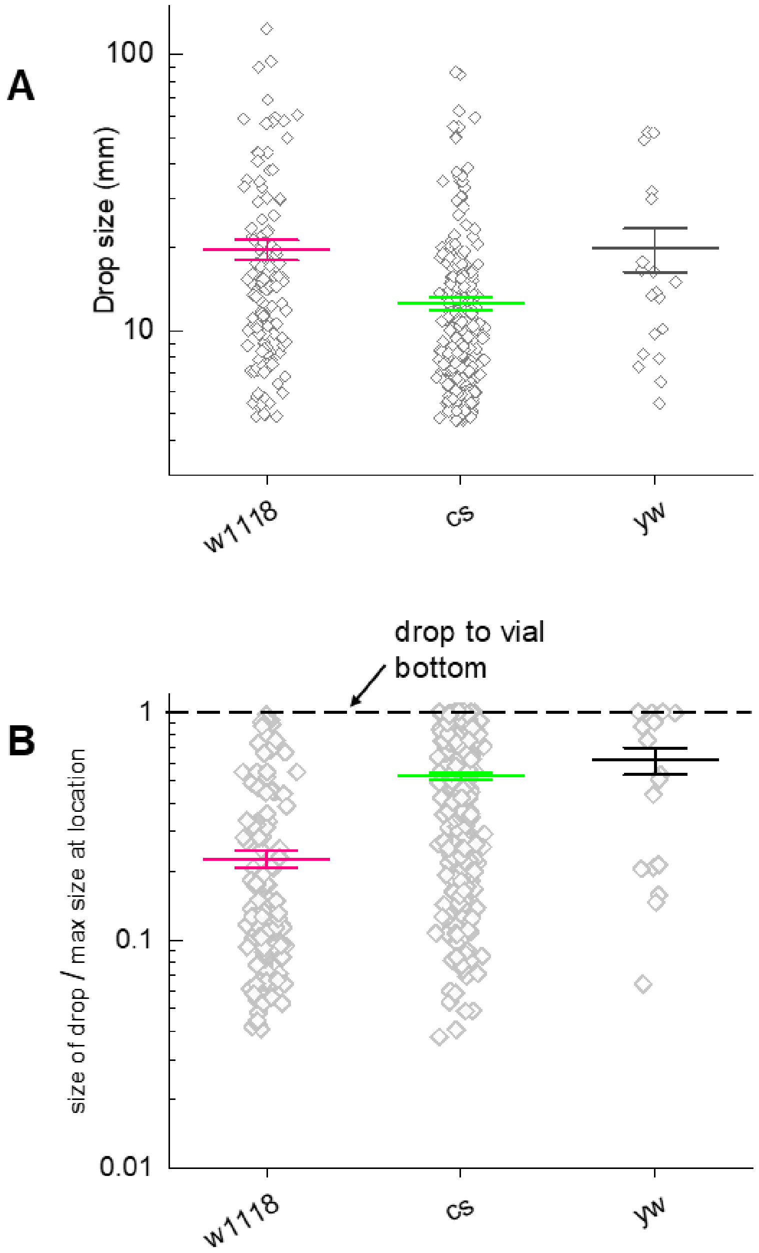

Fig. S5. A closer examination of drop statistics. N = 18 w^{1118}, 17 *CS*, and 21 *yw* from Figure 4. (A) Size of slips and falls of the three strains. w^{1118} and *yw* drop sizes are similar (P=0.92) but larger than *CS* (P=2x10^{-6} vs. w^{1118} and P=0.024 vs. *yw*). (B) Ratio of drop size to maximum possible drop from that height. The fractional drop size of w^{1118} flies is on average the smallest (P < 7x10^{-6}) while that of *CS* are moderately smaller than *yw* (P=0.5). Mean±standard errors are shown in both panels. P values are from Kruskal-Wallis null hypothesis test followed by a post hoc test.

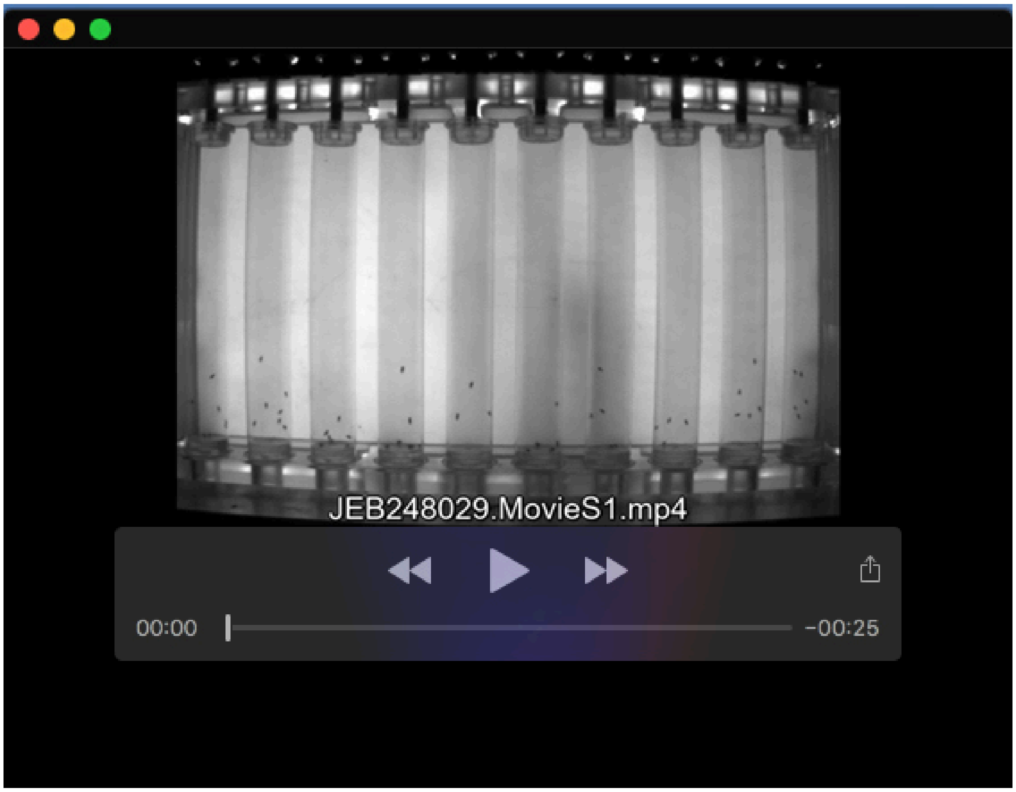

Movie 1. An example of a typical experimental trial video. A forward-facing 12 second recording of the vial mount from the automated apparatus showcasing 10 wild type cohorts. Vials 1-3 contain *yw* females (N=7 flies/vial), vial 4 contains *CS* females (N=6), vial 5 contains w^{1118} females (N=3), vial 6 contains w^{1118} males (N=5), vial 7 contains *CS* males (N=5), vials 8-10 contain *yw* males (N=4,6,5 respectively).

Journal of Experimental Biology • Supplementary information

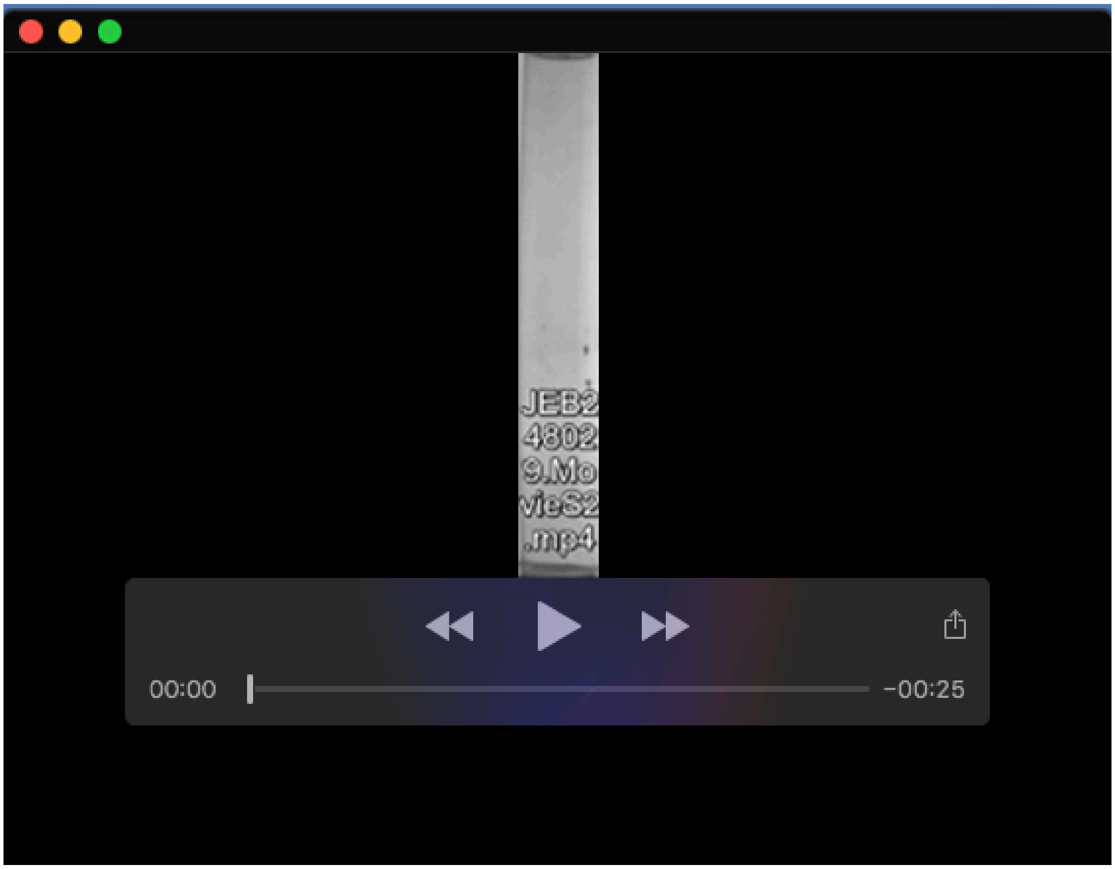

Movie 2. An example of a ROI video after video processing. From the original trial video (Movie 1), the user defined Region of Interest (ROI) is used to cut the 12 second video into individual vial videos. This example is vial 2, female *yw* flies (N = 7).

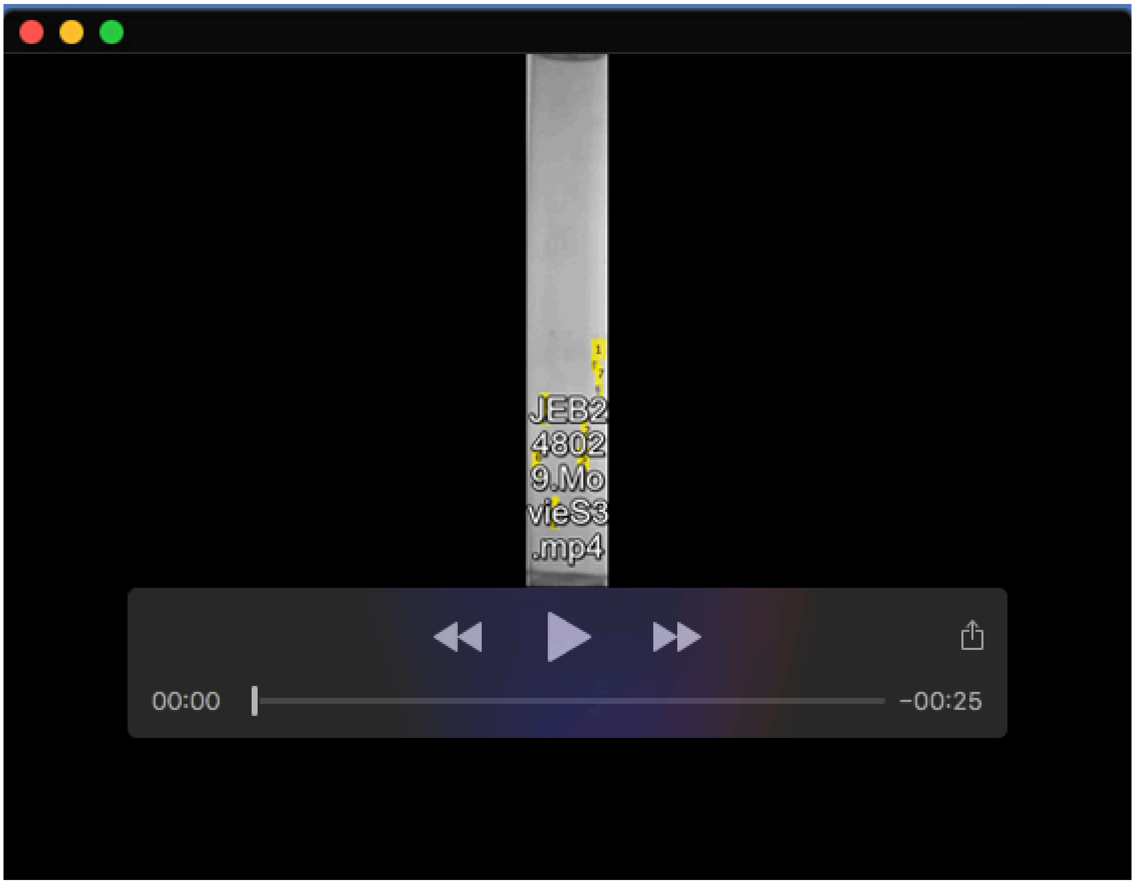

Movie 3. A real time visual representation of the output from the tracking algorithm. Using the same ROI video from Movie 2 (vial 2, *yw* females, N=7), per frame each fly is detected, shown by the yellow bounding boxes, and assigned a corresponding number attached by yellow flags. The centers of the bounding boxes in each video frame correspond to the coordinates saved in the algorithms output matrix. The video shows several collisions between flies. Analysis of a larger dataset of 79800 frames (266 flies x 10 seconds x 30 frames s^{-1}) detected 2762 collisions, amounting to ~ 3.5% of possible instances. Not every collision leads to switching in identities. As an upper estimate, track label swapping occurs in < 2% of the time.
